# Eicosapentaenoic acid/arachidonic acid ratio and weight loss during hospitalization for glycemic control among overweight Japanese patients with type 2 diabetes: a retrospective observational study

**DOI:** 10.1186/s12944-019-0983-x

**Published:** 2019-01-31

**Authors:** Shuhei Nakanishi, Hidenori Hirukawa, Masashi Shimoda, Fuminori Tatsumi, Kenji Kohara, Atsushi Obata, Seizo Okauchi, Tomoe Kinoshita, Junpei Sanada, Yoshiro Fushimi, Momoyo Nishioka, Yuki Kan, Akiko Tomita, Akiko Mashiko, Megumi Horiya, Yuichiro Iwamoto, Tomoatsu Mune, Kohei Kaku, Hideaki Kaneto

**Affiliations:** 1 Division of Diabetes, Metabolism and Endocrinology, Kawasaki Medical School, 577 Matsushima, Kurashiki, Okayama, 701-0192 Japan; 2 Department of Internal Medicine, Kawasaki Medical School, Okayama, Japan

**Keywords:** Eicosapentaenoic acid, Arachidonic acid, Body weight loss, Type 2 diabetes

## Abstract

**Background:**

The study aimed to examine the relationship between levels of serum eicosapentaenoic acid (EPA), arachidonic acid (AA), as well as EPA/AA ratio and weight loss during hospitalization in participants considered to be overweight, with type 2 diabetes.

**Methods:**

The study participants included 142 patients who were hospitalized for treatment of type 2 diabetes. We divided the participants into two groups depending on the achievenemt in reduction of bodyweight 3% or more during hospitalization and examined the relationship between serum levels of EPA and AA, as well as ratio of EPA/AA on admission and effectiveness of weight loss under strict dietary therapy during hospitalization, using Cox proportional hazard models.

**Results:**

After adjustment was made for several confounders, the hazard ratio of effective weight loss for logarithmical serum EPA was 1.59 (95% CI 1.02–2.49, *P* = 0.04) and for logarithmical EPA/AA ratio 1.64 (1.03–2.61, *P* = 0.04), whereas the hazard ratio for effective weight loss for logarithmical serum AA was 1.11 (0.45–2.78, *P* = 0.82). In addition, after dividing EPA/AA ratio and serum EPA into quartiles based on participant number, the hazard ratio for the highest quartile of EPA/AA ratio was 2.33 (1.14–4.77, *P* = 0.02), and for the highest quartile of serum EPA 1.60 (0.80–3.19, *P* = 0.18) compared with the lowest quartile.

**Conclusion:**

These results suggest the possibility that EPA is involved in bodyweight change under a caloric-restriction regimen. In addition, EPA/AA ratio was found to be a better predictor of medical intervention for weight loss among overweight patients with type 2 diabetes, compared with serum EPA level.

## Background

Obesity is a metabolic disorder whose prevalence has been increasing dramatically in most developed countries over the last 30 years. In fact, in 2015, Global Burden of Disease Study data estimated that 603.7 million adults across the globe were obese [1]. Obesity is associated with an increased risk of developing chronic morbidities (hypertension, insulin resistance, dyslipidemia), which constitute the major components of metabolic syndrome. For treatment of type 2 diabetes, intensive bodyweight management based on strict dietary therapy is an important strategy, with remission of the disease a conceivable outcome [2]. Against these circumstances, the 2013 AHA/ACC/TOS guidelines recommend sustained weight loss of more than 3%, because that level would likely result in clinically meaningful reductions in blood glucose and HbA1c [3]. Indeed, in Japanese overweight and obese subjects, weight reduction of at least 1–3% was reported to improve HbA1c level [4].

Fish and omega-3 polyunsaturated fatty acid (n-3 PUFA) intake is thought to prevent cardiovascular disease [5, 6] and type 2 diabetes [7], a hypothesis supported by an animal experiment showing favorable effects of n-3 PUFA, which are abundant in fish, on insulin resistance [8]. Several studies explored the effects of oral intake of n-3 PUFA (either fish or fish oil) on bodyweight and body composition in human study participants. In randomized controlled trials, available evidence suggests conflicting results about the effects of n-3 PUFA on bodyweight and body composition. Two recent studies examined the effects of a combination of n-3 PUFA and dietary caloric restriction in obese volunteers. Parra et al. [9] showed that satiety was increased after consumption of n-3 PUFA-enriched meals. Thorsdottir et al. [10] showed greater bodyweight loss and waist circumference reduction in male patients receiving fish or fish oil.

Such results from small numbers of trials, however, do not provide enough robust data to conclude that n-3 PUFA can modify, and especially reduce, bodyweight. Marked differences in experimental design, intervention type and duration, baseline characteristics of participants (degree of obesity, associated conditions, etc.), attrition rate, and doses of n-3 PUFA make the results inconclusive and, in some cases, discordant. It is worth noting, therefore, that a major limitation of these trials is the absence of appropriate and precise control of caloric intake to assess weight loss.

The aim of this retrospective study was to examine serum levels of EPA and AA, as well as the ratio of EPA/AA on admission and investigate their association with the extent of weight loss more than 3% by strict dietary therapy during hospitalization for treatment of type 2 diabetes, under the hypothesis that n-3 PUFA may be involved in and regulate bodyweight changes.

## Methods

### Study population and patient preparation

**Fig. 1:**
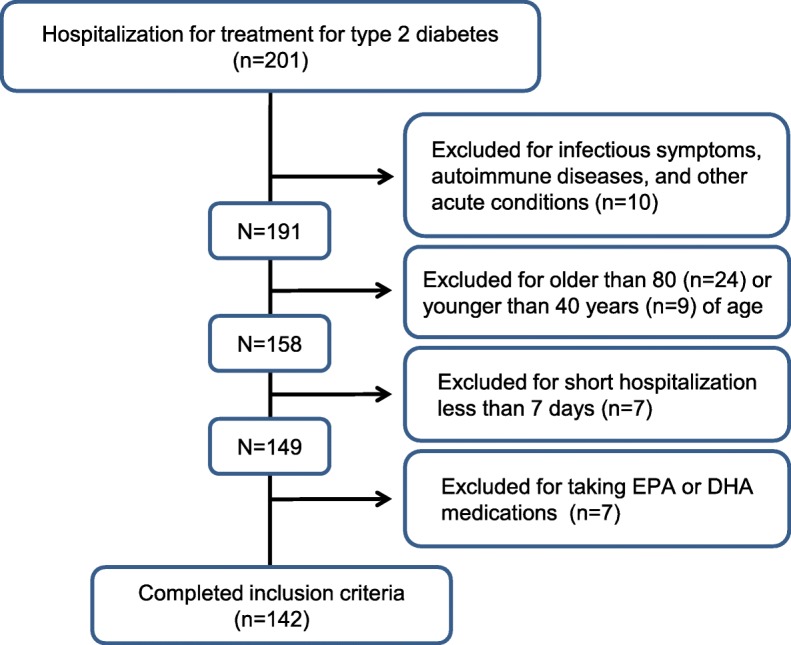
The flow chart of the study participants

The study participants comprised 82 men and 60 women who had been hospitalized for treatment of type 2 diabetes at Kawasaki Medical School Hospital at some point between January 2016 and March 2018. As the inclusion criteria, all patients with type 2 diabetes who were hospitalized for glycemic control were included. All 201 hospitalized patients underwent a medical interview and examinations on admission to ensure that all the participants were free of infectious symptoms, autoimmune diseases, and other acute conditions. Participants younger than 40 years of age and older than 80 were excluded to control generational disparities. In addition, participants hospitalized for a shorter period than seven days or currently taking EPA or DHA medications were excluded from the study (Fig. 1). At the beginning of hospitalization, participant height and weight were measured in the standing position. Body mass index (BMI) was calculated, both on admission and at discharge, for each participant as body weight (kg) divided by the square of standing height (m). Blood and urine samples were taken from all participants the day following admission after an overnight fast. Glycoalbumin (GA) was measured by using an enzymatic method and HbA1c with the HPLC method. Measurement of participants’ EPA and AA levels was outsourced to SRL (Tokyo, Japan). In brief, plasma lipids were extracted by Folch’s procedure, and fatty acids (tricosanoicacid, C23:0, as the internal standard) were methylated with boron trifluoride and methanol. The methylated fatty acids were then determined and analyzed using a capillary gas chromatograph (Shimadzu GC-2010, Kyoto, Japan). Microangiopathy in all participants was evaluated as follows: diabetic retinopathy was diagnosed by ophthalmologists [11]; diabetic nephropathy was classified from normal to dialysis stage according to the classification of diabetic nephropathy 2014 [12], in view of eGFR status and albuminuria; and diabetic neuropathy was assessed by the physician in charge based on the abbreviated criteria by the Diabetic Neuropathy Study Group in Japan [13]. In addition, as possible confounding factors contributing to change of weight loss during hospitalization, the following were used: duration of diabetes, medication for hypertension and/or dyslipidemia and BMI on admission, using sodium-glucose cotransporter 2 (SGLT2) inhibitors or glucagon-like peptide-1 (GLP-1) analogues, and caloric intake during hospitalization. All study participants were restricted from snacking between three mealtimes, and caloric intake from meals were controlled and calculated during hospitalization by the physicians in charge based on each of the participant’s ideal body weight. Medication was chosen by the physician in charge together with the diabetes health-care team based on a patient-centered approach considering the best available evidence in terms of benefits, risks, patient values, preferences, and context in time, not only target glucose and HbA1c levels. The hospital ethics committee approved the study protocol, and information on the study was provided to the public via the Internet, in place of obtaining informed consent from each patient (No. 3115).

### Statistical analysis

The data are expressed as the mean and standard deviation. Continuous variables at the start of the study were compared using an age and gender-adjusted analysis of covariance (ANCOVA) for comparisons between categories of clinical characteristics at baseline. Since the BMI, EPA, and AA data were not normally distributed, these data were analyzed after logarithmic transformation. To test the effectiveness of hospitalization for weight loss, all participants were measured for the percentage of decrease in bodyweight based on bodyweight at admission and at discharge ((bodyweight at discharge-bodyweight at admission)/bodyweight at admission) and were designated as succeeding in losing weight if 3% or more of bodyweight was lost during hospitalization [3, 4]. This category was designated the dependent variable (1, effective weight loss of at least 3%; 0, ineffective weight loss of less than 3%). To clarify the significance of serum EPA or AA levels and EPA/AA ratio as predictors of effectiveness of weight loss, in addition to the numerical concentrations or ratio itself as continuous variables, their concentrations and the ratio were divided into quartiles based on subject number (< 37.1, 37.1–54.0, 54.1–74.0 and > 74.0 μg/mL in EPA; < 172.4, 172.4–199.5, 199.6–237.3, and > 237.3 μg/mL in AA; and < 0.18, 0.18–0.24, 0.25–0.39 and > 0.39 in EPA/AA ratio); numerical and quartile-specific hazard ratios were estimated with a Cox proportional hazards model. Participant numbers were 37, 34, 36, and 35, respectively, for the EPA category; 36, 35, 36, and 35, respectively, for the AA category; and 37, 35, 36, and 34, respectively, for the EPA/AA ratio category. To confirm the effect of levels of EPA, AA, and EPA/AA ratio at admission on the extent of weight loss during hospitalization, a Cox proportional hazards model was used, after adjustment was made for the confounders age, gender, duration of diabetes, medication for SGLT-2 inhibitors and GLP-1 RA, reduced caloric intake during hospitalization, and HbA1c and BMI on admission. There were no missing data concerning these confounders. The statistical analyses were performed using the SAS software program (version 8 for Windows).

## Results

### Clinical characteristics of the study participants

Serum EPA and AA levels, as well as EPA/AA ratio did not differ between men and women, and therefore all data for men and women were combined. Mean age, duration of type 2 diabetes at admission, and hospitalization period were 62.2 ± 10.6 years, 12.6 ± 9.4 years, and 12.2 ± 2.8 days, respectively. Mean HbA1c, serum EPA and AA levels, as well as EPA/AA ratio were 9.8 ± 2.1%, 60.1 ± 32.0 μg/mL, 208.5 ± 54.8 μg/mL, and 0.31 ± 0.19, respectively. During the hospitalization period, percent change of bodyweight loss was lost 3.6 ± 3.8%. BMI was reduced from 26.3 ± 5.9 kg/m^2^ to 25.3 ± 5.5 kg/m^2^ by personalized caloric restriction. GA decreased from 25.4 ± 7.9% to 21.9 ± 5.6% during hospitalization. Table 1 indicates the clinical characteristics at baseline.

**Table 1:** Clinical characteristics of study subjects

	All subjects	Lowest quartile	Low quartile	High quartile	Highest quartile
M/F (n)	82/60	19/18	18/17	23/13	22/12
Age (years)	62.2 ± 10.6	55.6 ± 9.6	59.3 ± 11.8	65.5 ± 8.6†	68.9 ± 6.5†
Duration of type 2 diabetes (years)	12.6 ± 9.4	10.1 ± 8.7	11.3 ± 8.4	13.7 ± 9.7	15.5 ± 10.2
Hospital stay (days)	12.2 ± 2.8	13.0 ± 3.5	11.7 ± 2.4*	12.4 ± 2.9	11.8 ± 2.2†
Diet calorie (kcal)	1546 ± 183	1576 ± 205	1529 ± 171	1544 ± 176	1532 ± 181
BMI on admission (kg/m^2^)	26.3 ± 5.9	27.2 ± 5.6	26.8 ± 6.2	25.2 ± 6.7	26.1 ± 4.8
Body weight on admission (kg)	68.6 ± 17.0	72.5 ± 18.0	70.0 ± 18.0	64.5 ± 16.4	67.1 ± 15.0
HbA1c on admission (%)	9.8 ± 2.1	10.6 ± 2.4	9.4 ± 1.8*	9.8 ± 2.0*	9.4 ± 2.0
GA on admission (%)	25.4 ± 7.9	26.0 ± 7.7	23.2 ± 7.5	27.8 ± 9.3	24.4 ± 6.5
BMI at discharge (kg/m^2^)	25.3 ± 5.5	26.3 ± 5.3	26.0 ± 6.0	23.9 ± 6.1	25.2 ± 4.6
Body weight at discharge (kg)	66.0 ± 16.2	70.2 ± 17.2	67.9 ± 17.4	61.1 ± 16.6	64.7 ± 14.3
GA at discharge (%)	21.9 ± 5.6	22.1 ± 5.0	19.9 ± 5.4	24.2 ± 6.9	21.3 ± 4.5
EPA (μg/mL)	60.1 ± 32.0	31.8 ± 11.0	47.9 ± 13.6†	63.9 ± 17.5†	99.2 ± 32.8†
AA (μg/mL)	208.5 ± 54.8	232.0 ± 58.0	223.3 ± 63.1	198.0 ± 43.9*	178.7 ± 33.7†
EPA/AA	0.31 ± 0.19	0.14 ± 0.03	0.22 ± 0.02†	0.32 ± 0.05†	0.57 ± 0.19†
Retinopathy (N/S/P)	96/29/17	22/10/5	24/4/7	26/8/2	24/7/3
Neuropathy (n)	54	10	10	19	15
Nephropathy (Stage 1/2/3/4)	91/36/13/2	22/13/1/1	20/7/8/0	26/9/1/0	23/7/3/1
Treatment for diabetes (n)					
Insulin/SU/Glinides/TZD	44/39/10/31	11/11/1/7	10/5/5/6	13/8/2/6	10/15/2/12
BG/α-GI/DPP-4I	66/26/73	25/6/16	13/7/20	10/6/17	18/7/20
SGLT2I/GLP1RA	19/13	8/7	6/1	2/3	3/2
Treatment for dyslipidemia (n)	118	23	35	26	34
Treatment for hypertension (n)	69	19	12	15	23

Data are shown as mean ± SD. *: *P* < 0.05 compared to category of lowest quartile adjusted for age and sex except age. †: *P* < 0.01 compared to category of lowest quartile adjusted for age and sex except age. *BMI* body mass index, *GA* glycoalbumin, *EPA* eicosapentaenoic acid, *AA* arachidonic acid, *N/S/P* none/simple/pre or proliferative, *SU* sulfonylureas, *TZD* thiazolidinedione, *BG* biguanide, *α-GI* alpha-glucosidase inhibitors, *DPP-4I* dipeptidyl peptidase-4 inhibitors, *SGLT2I* sodium-glucose linked transporter 2 inhibitors, *GLP1RA* glucagon-like peptide 1 receptor agonist

### Impact of serum EPA and AA levels, as well as EPA/AA ratio on effective loss of bodyweight during hospitalization

**Fig. 2:**
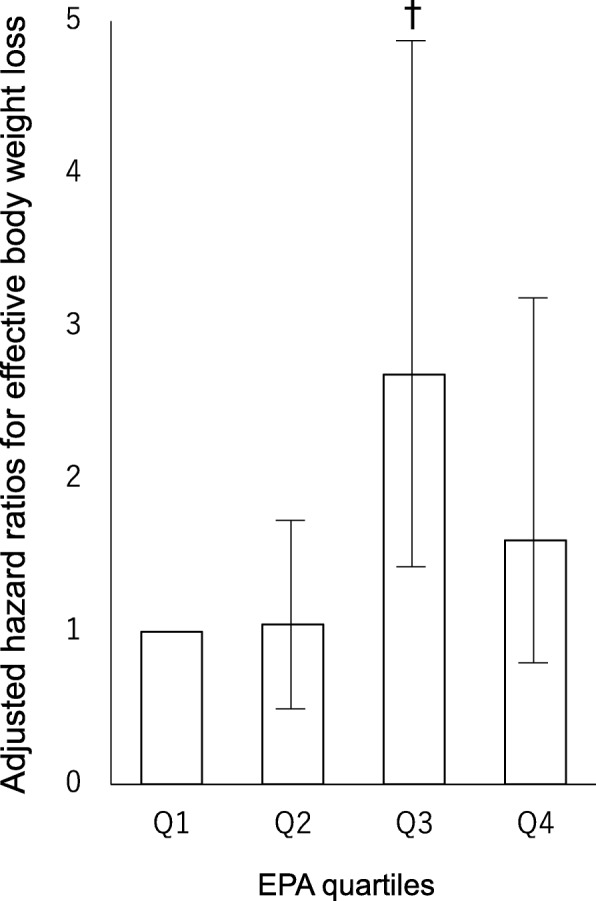
Adjusted hazard ratios for effective body weight loss among patients with type 2 diabetes. The participants were divided into quartiles by serum EPA level on admission. †*P* < 0.01 compared to the lowest quartile

A Cox proportional hazards model was used in analysis based on serum EPA levels, after adjustment was made for the previously indicated confounders, and as a result the hazard ratio for effective weight loss during hospitalization was 1.59 (95% CI 1.02–2.49, *P* = 0.04). In addition, a Cox proportional hazards model was used in analysis including four categories based on serum EPA levels, after adjustment was made for the previously mentioned confounders, and as a result the hazard ratios by increasing quartile of serum EPA levels were 1.0, 1.05 (0.55–2.00, *P* = 0.89), 2.69 (1.46–4.95, *P* = 0.002), and 1.60 (0.80–3.19, *P* = 0.18), respectively (*P* = 0.04 for trend) (Fig. 2).

**Fig. 3:**
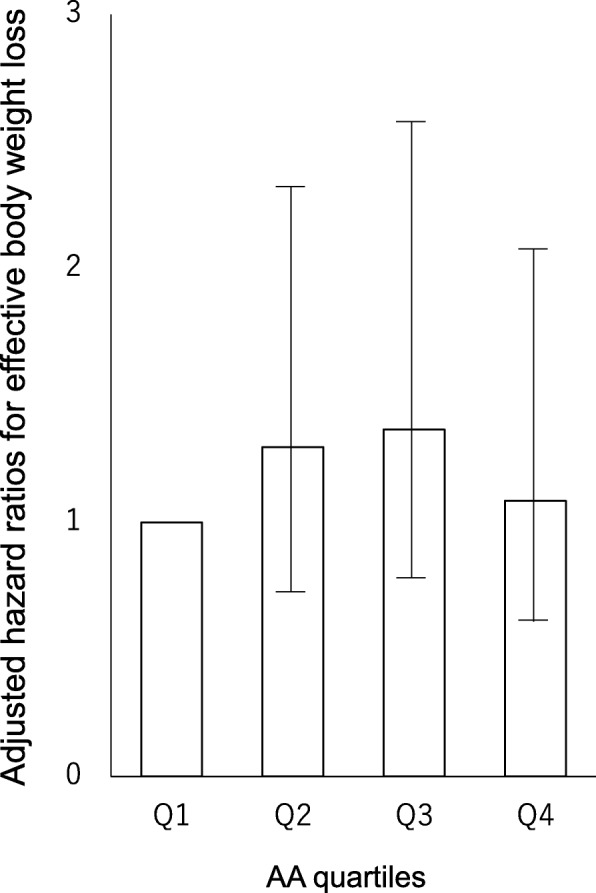
Adjusted hazard ratios for effective body weight loss among patients with type 2 diabetes. The participants were divided into quartiles by serum AA level on admission

The same model was used for serum AA levels, after adjustment was made for the same confounders, and the hazard ratio for effective weight loss during hospitalization was 1.11 (0.45–2.78, *P* = 0.82). In addition, the same model was used in analysis including four categories based on serum AA levels, after adjustment was made for the same confounders, and as a result the hazard ratios by increasing quartile of serum AA levels were 1.0, 1.30 (0.70–2.40, *P* = 0.41), 1.37 (0.73–2.57, *P* = 0.33), and 1.09 (0.55–2.14, *P* = 0.81), respectively (*P* = 0.71 for trend) (Fig. 3).

**Fig. 4:**
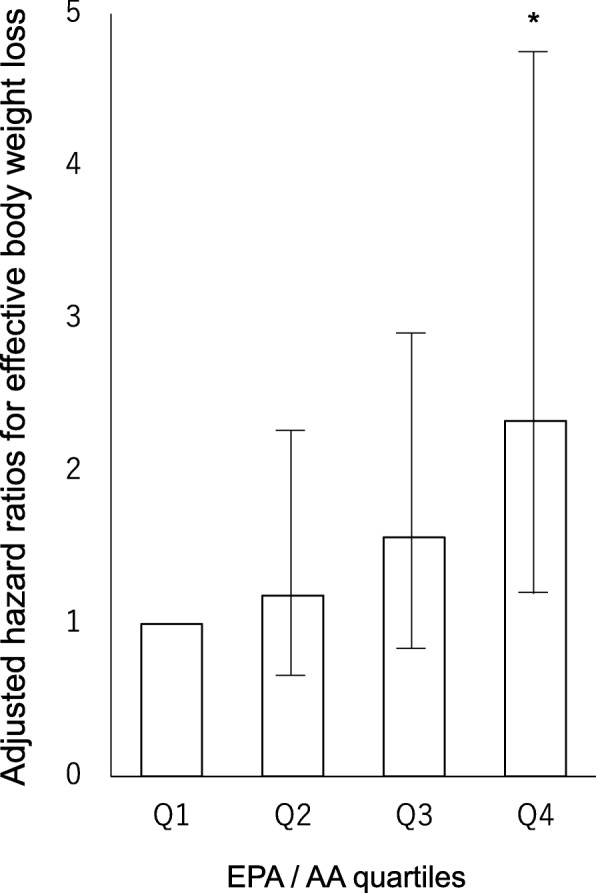
Adjusted hazard ratios for effective body weight loss among patients with type 2 diabetes. The participants were divided into quartiles by EPA/AA ratio on admission. *P < 0.05 compared to the lowest quartile

The same model was used in analysis based on EPA/AA ratio, after adjustment was made for the same confounders, and as a result the hazard ratio for effective weight loss during hospitalization was 1.64 (1.03–2.61, *P* = 0.04). In addition, the same model was used in analysis including four categories based on EPA/AA ratio, after adjustment was made for the same confounders, and as a result the hazard ratios by increasing quartile of EPA/AA ratio were 1.0, 1.19 (0.61–2.30, *P* = 0.61), 1.57 (0.82–2.99, *P* = 0.17), and 2.33 (1.14–4.77, *P* = 0.02), respectively (*P* = 0.02 for trend) (Fig. 4).

### Comparisons among the study participants divided into quartiles by EPA/AA ratio

Since a strong relationship between EPA/AA ratio and weight loss was observed, as described above, the study participants were divided into quartiles by EPA/AA ratio. Table 1 indicates the clinical characteristics of participants in each quartile. Compared to the patients in the lowest quartile, the patients in the high and highest quartiles were significantly older, after adjustment was made for sex. Compared with the patients in the lowest quartile, the hospitalization periods of the patients in the low and highest quartiles were significantly shorter, after adjustment was made for age and sex. In contrast, among the four categories, there were no differences in duration of diabetes, BMI, body weight and GA on admission and at discharge, or in reduced caloric intake during hospitalization, after adjustment was made for sex and age.

## Discussion

In this retrospective observational study, serum levels of EPA and EPA/AA ratio were significantly associated with weight loss by caloric restriction during hospitalization among Japanese patients considered to be overweight with type 2 diabetes. To our knowledge, this is the first study and analysis to examine the effects of serum n-3 PUFA levels and bodyweight change under strict caloric restriction. The result suggests that having high serum levels of n-3 PUFA may potentially effectively augment the effect of intervention such as diet therapy among overweight patients with type 2 diabetes.

High serum levels of n-3 PUFA, such as EPA, may assist with bodyweight and body fat reduction by multiple mechanisms. The principal hypothesis of this study was that high serum levels of n-3 PUFA would result in an increase in these fatty acids at the target tissue level such as fat and muscle tissues, leading to effective improvement in weight loss under caloric control. The results from this study support this hypothesis. In addition, it was reported that higher plasma levels of n-3 fatty acids are associated with lower BMI, waist circumference, and hip circumference among non-diabetes study participants [14], as well as with lower visceral fat among male patients with type 2 diabetes [15]. Another study involving 120 normal-weight and overweight adolescents found that overweight adolescents had lower total n-3 PUFA concentrations compared with normal-weight adolescents, independent of body fat and fat distribution [16]. These findings and our results combined suggest that these fatty acids may potentially contribute to a healthy bodyweight and the prevention of abdominal adiposity among overweight participants.

The results from our study are biologically plausible, because several mechanisms underlying the association between n-3 PUFA and obesity have been shown in previous studies. One possibility is that n-3 PUFA could increase basal fat oxidation, which may in turn reduce fat mass [17, 18]. In addition, n-3 PUFA increase mitochondrial oxidative capacity in white adipose tissues [19] and skeletal muscle, possibly through UCP-3 up-regulation [20]. These results indicate that basic n-3 PUFA concentrations may hold the key to thermogenesis, which may provide a defense against obesity. Furthermore, one recent study has shown that n-3 PUFA intake increases postprandial satiety in overweight and obese individuals during weight loss [9]. Study participants with high levels of serum n-3 PUFA may thus be able to control their appetite during hospitalization, leading to successful weight loss among overweight patients with type 2 diabetes.

Another reason for these results may be explained by the effects of GLP-1, which could lead to weight loss and reduction in appetite [21]. GLP-1 is likely secreted upon n-3 PUFA administration, a hypothesis demonstrated in some examples of basic research [22, 23]. Indeed, serum EPA levels are related to the effect of dipeptidyl-peptidase IV (DPP-4) inhibitor, which decreases blood glucose levels through increasing circulating GLP-1 levels, in Japanese patients with type 2 diabetes [24].

The other, and most plausible, reason for these results is that the anti-inflammatory potential of EPA [25, 26] might directly accelerate weight loss under caloric restriction through the inhibition of adipose tissue inflammation associated with obesity [27]. In rodent models of obesity, EPA was found to increase the production of adiponectin, possibly through the attenuation of inflammatory changes and the reduction of adipocyte cell size [28]. Thus, the association between the EPA/AA ratio and weight loss in this study might have been, at least partially, explained by the following reason: It is likely that the high serum level of EPA, relative to the level of AA, leads to a reduction of adipose tissue weight through the anti-inflammatory effects of EPA. In other words, anti-inflammation may induce alterations in the expression of genes involved in the regulation of fat oxidation in adipose, liver, cardiac, intestinal, and skeletal muscle tissues, and in the regulation of adipogenesis in adipose tissue. These effects on gene expression favor enhanced fat oxidation and reduced fat deposition.

There are a variety of approaches to investigating the effects of n-3 PUFA on bodyweight, body composition, and energy intake in human interventions that use different types of fish and varying levels of fish oil content, particularly EPA and DHA [29–31]. However, to draw a conclusion about this issue is difficult due to the variety of caloric restriction programs used in different studies, but it will be important to verify whether increased n-3 PUFA concentrations prior to interventions would aid in weight loss and ameliorate obesity-related metabolic dysfunctions. Considering the results of this study, the effect of n-3 PUFA on bodyweight control might be effective under strict caloric restriction, but this study did not have an interventional design. Serum n-3 PUFA levels in this study were supplied from daily food intake before admission, mainly from fish oils, it is assumed. The possibility thus remains that the results of this study were derived from other ingredients besides fish oil. Further study to clarify the role of n-3 PUFA in obesity management is therefore warranted.

The present study has several limitations. First, it was a retrospective observational study with a limited study population. The observational period was also limited. In addition, we examined only the patients with type 2 diabetes. Therefore, it was difficult to generalize these results and conclude the effects of n-3 PUFA with any certainty. In particular, only EPA among the different forms of n-3 PUFA was assessed. The effects of other n-3 PUFA such as DHA were not evaluated. Second, diabetes medications taken during hospitalization were not considered in this study. Any medication used was chosen by the physician in charge, taking a patient-centered approach that considered the best available evidence, benefits, risks, patient values, preferences, and context in time, and was not chosen only by targeting HbA1c level in all patients. Third, patients with a low EPA/AA ratio were young compared to patients with a high EPA/AA ratio, although inclusion criteria were made for patients aged from 41 to 79 years and statistically adjusted for age in our study. Some unknown factors related to age or generation might have affected the results besides n-3 PUFA factors. Lastly, we did not evaluate habitual and comorbid factors such as smoking status, activity of daily lives, and fish intake not only before but also during hospitalization.

## Conclusions

In conclusion, we found that overweight type 2 diabetes patients with high serum levels of EPA or a high EPA/AA ratio had an enhanced ability to reduce bodyweight effectively under a caloric-restriction regimen during hospitalization. The results of this observational study suggest that maintaining high serum levels of n-3 PUFA, such as EPA, may play an important role in preventing weight gain and increasing weight loss when used concomitantly with a structured weight-loss program in type 2 diabetes patients.

## Abbreviations

AA: Arachidonic acid
ANCOVA: Analysis of covariance
BMI: Body mass index
CI: Confidence interval
DPP-4: Dipeptidyl-peptidase IV
eGFR: estimated glomerular filtration rate
EPA: Eicosapentaenoic acid
GA: Glycoalbumin
GLP-1: Glucagon-like peptide-1
n-3 PUFA: Omega-3 polyunsaturated fatty acid
SGLT2: Sodium-glucose cotransporter 2
UCP: Uncoupling protein
